# Supplementary material for: Boosting the catalysis of gold by O2 activation at Au-SiO2 interface
Source: Nat Commun. 2020 Jan 28;11:558. doi: 10.1038/s41467-019-14241-8 (PMC6987105; doi:10.1038/s41467-019-14241-8)
Supplement: Supplementary file 1 — Supplementary Information [file 41467_2019_14241_MOESM1_ESM.pdf]

Supplementary Information

Boosting the catalysis of gold by O_2 activation at Au-SiO_2 interface

Yunlai Zhang et al.

Supplementary Method

Method of micro-kinetic study

The micro-kinetic model was constructed by numerically solving the differential equations based on steady state approximation. In brief, the reaction rate constant (k_r) can be derived from the transition state theory (the Eyring equation).

$$k_{\mathrm{r}} = \frac{k_B T}{h} \exp\left(\frac{-E_a}{k_B T}\right) \quad (1)$$

where k_B, h, T and E_a represent the Boltzmann constant, Planck constant, absolute temperature and the activation energy of the reaction. The rate constant of adsorption (k_{ads}) was calculated by the following:

$$k_{\mathrm{ads}} = \frac{S \cdot P \cdot A}{\sqrt{2\pi m k_B T}} \qquad (2)$$

where S, P, A and m are the sticking coefficient, partial pressure of the adsorbed species, the area of the adsorption site, and the molecular mass of adsorbed species respectively. The value of S is assumed to one for all the species, since it is not very sensitive to the kinetic results.

The rate constant of desorption was calculated by the expression,

$$k_{\mathrm{des}} = \frac{k_B T^3}{h^3} \frac{A(2\pi m k_B)}{\sigma\theta_{rot}} \exp\left(\frac{-E_{des}}{k_B T}\right) \quad (3)$$

Where σ, θ_{rot} and E_{des} are the symmetry number, the characteristic temperature for rotation of adsorbed species, and the activation energy of desorption. The steady state approximation is adopted, assuming that the coverage changes of surface species are zero. The degree of thermodynamic rate control $X_{RC,i}$ of step i, which can qualitatively determine the significance of single step i in the overall reaction, is calculated as following,

$$X_{RC,i} = \frac{k_i}{r}\left(\frac{\partial r}{\partial k_i}\right)_{k_{j\neq i},K_i} = \left(\frac{\partial \ln r}{\partial \ln k_i}\right)_{k_{j\neq i},K_i} \qquad (4)$$

Where the partial derivative is taken holing constant for the rate constant k_j ($j \neq i$) and equilibrium constant K_i.

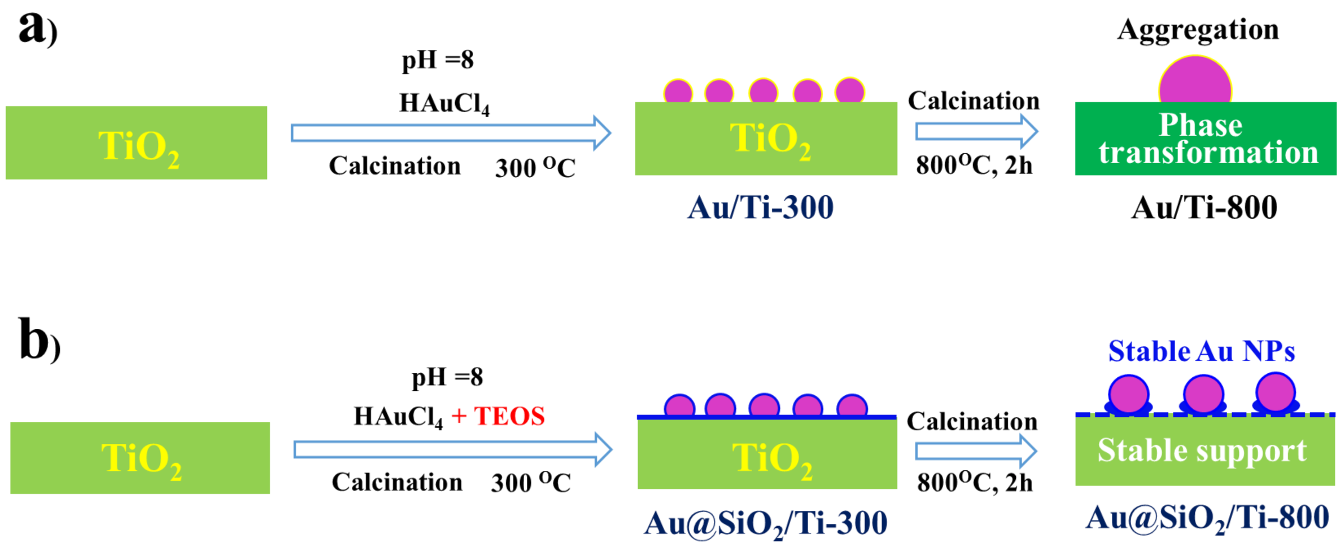

Supplementary Figure 1. Scheme of the synthetic strategy for the preparation of different catalysts. a) Au/Ti-T; b) Au@SiO_2/Ti-T.

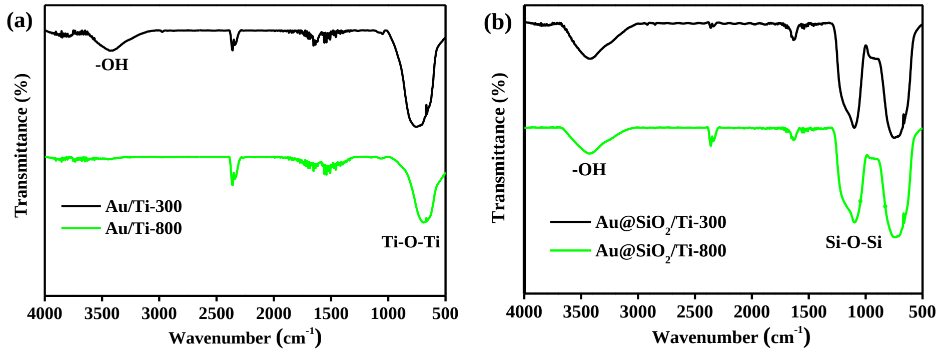

Supplementary Figure 2. FT-IR spectra of different catalysts. (a) Au/Ti-T; (b) Au@SiO_2/Ti-T.

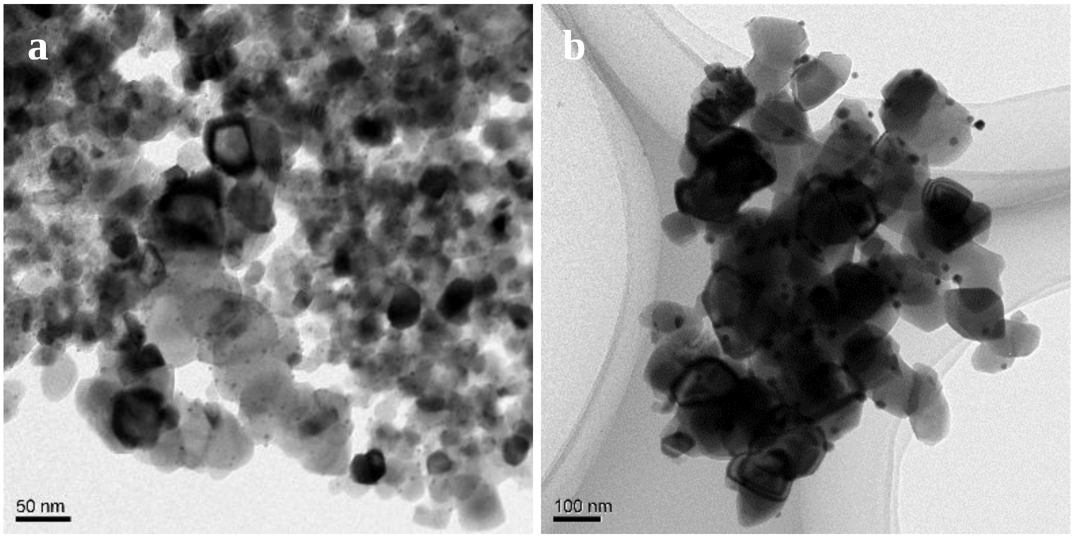

Supplementary Figure 3. TEM images of different catalysts. (a) Au/Ti-300; (b) Au/Ti-800.

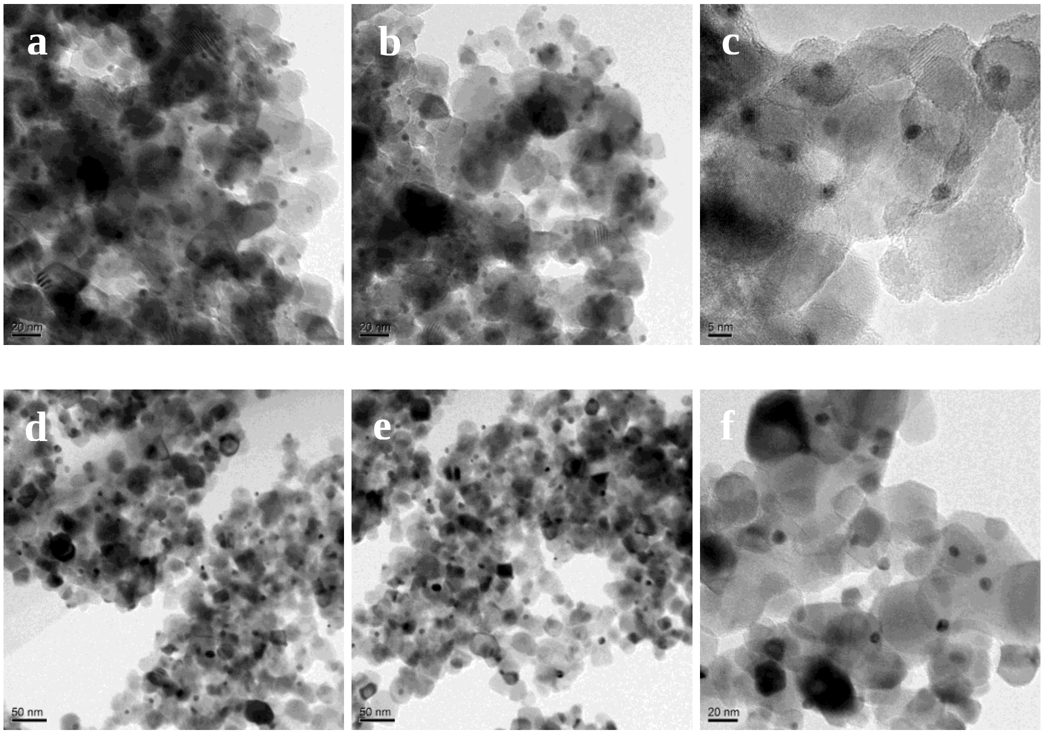

Supplementary Figure 4. TEM images of different catalysts. (a-c) Au@SiO_2/Ti-300; (d-f) Au@SiO_2/Ti-800.

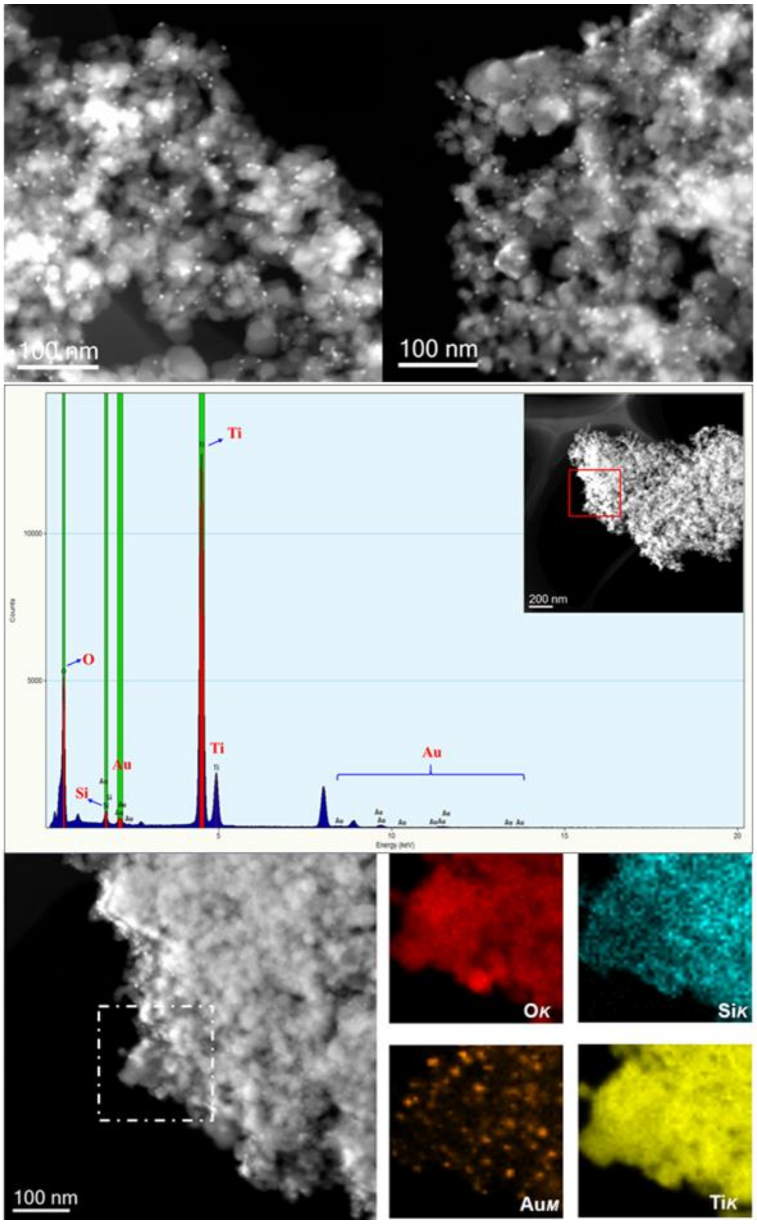

Supplementary Figure 5. HAADF-STEM and EDS mapping images of Au@SiO_2/Ti-300.

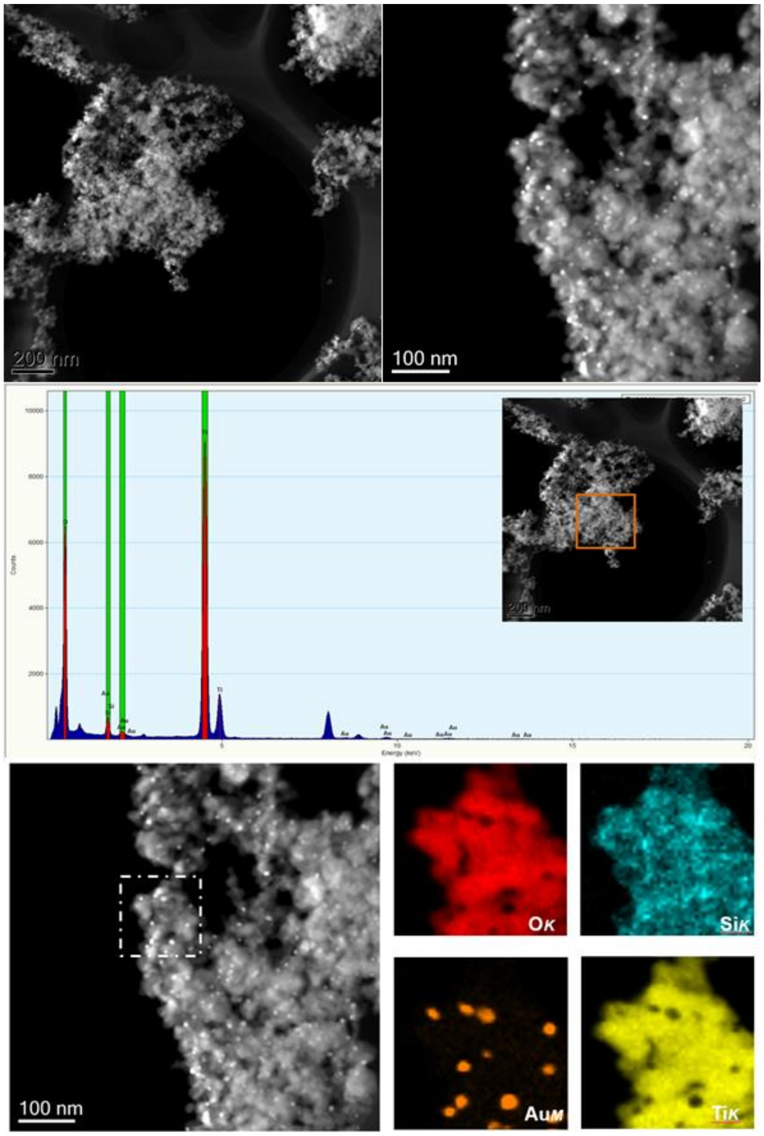

Supplementary Figure 6. HAADF-STEM and EDS mapping images of Au@SiO_2/Ti-800.

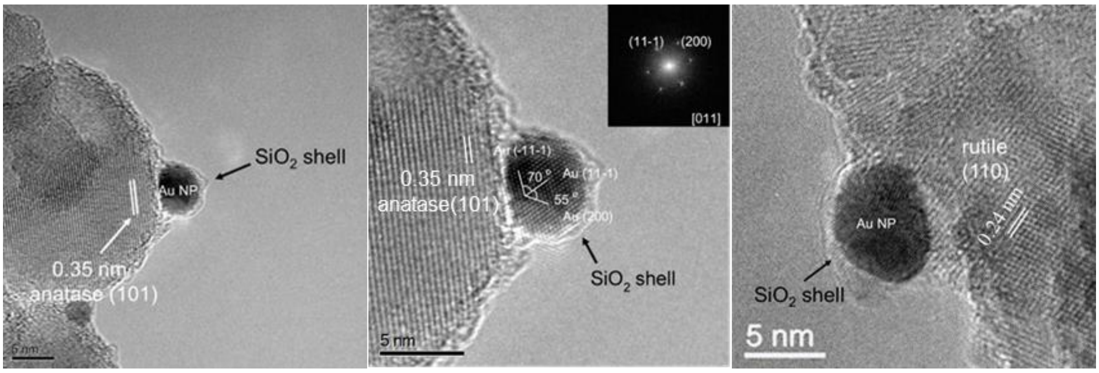

Supplementary Figure 7. HRTEM images of Au@SiO_2/Ti-300.

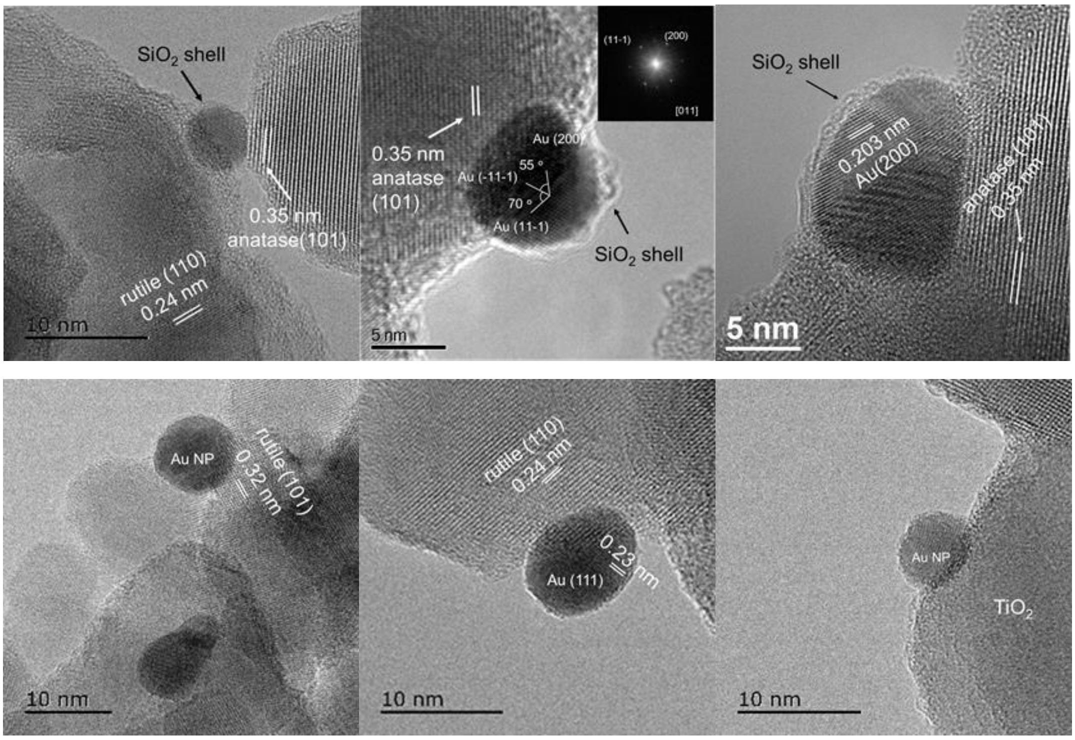

Supplementary Figure 8. HRTEM images of Au@SiO$_2$/Ti-800.

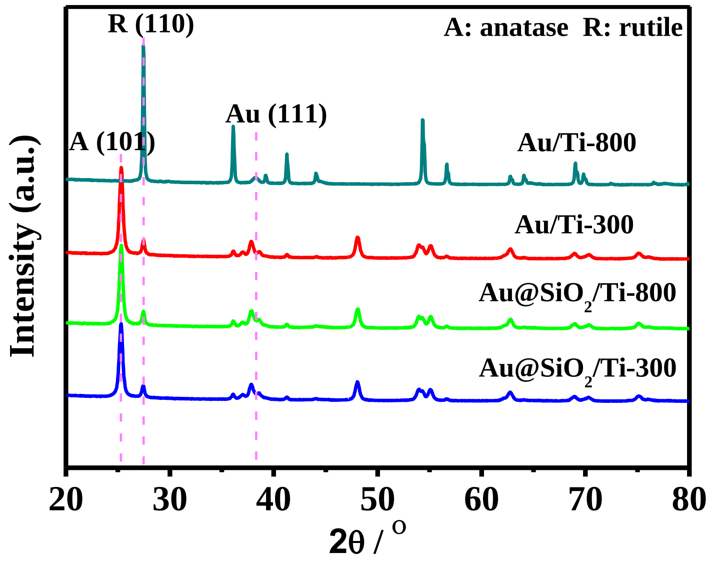

Supplementary Figure 9. Structural characterization of Au/Ti-T and Au@SiO_2/Ti-T catalysts annealed at temperatures of 300 and 800 °C, respectively.

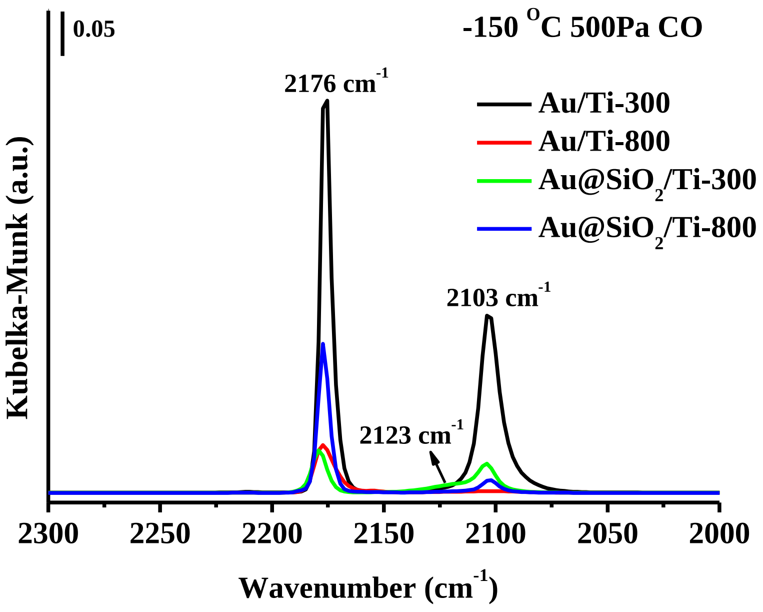

Supplementary Figure 10. DRIFT spectra of CO adsorption on various gold catalysts at -150 ºC.

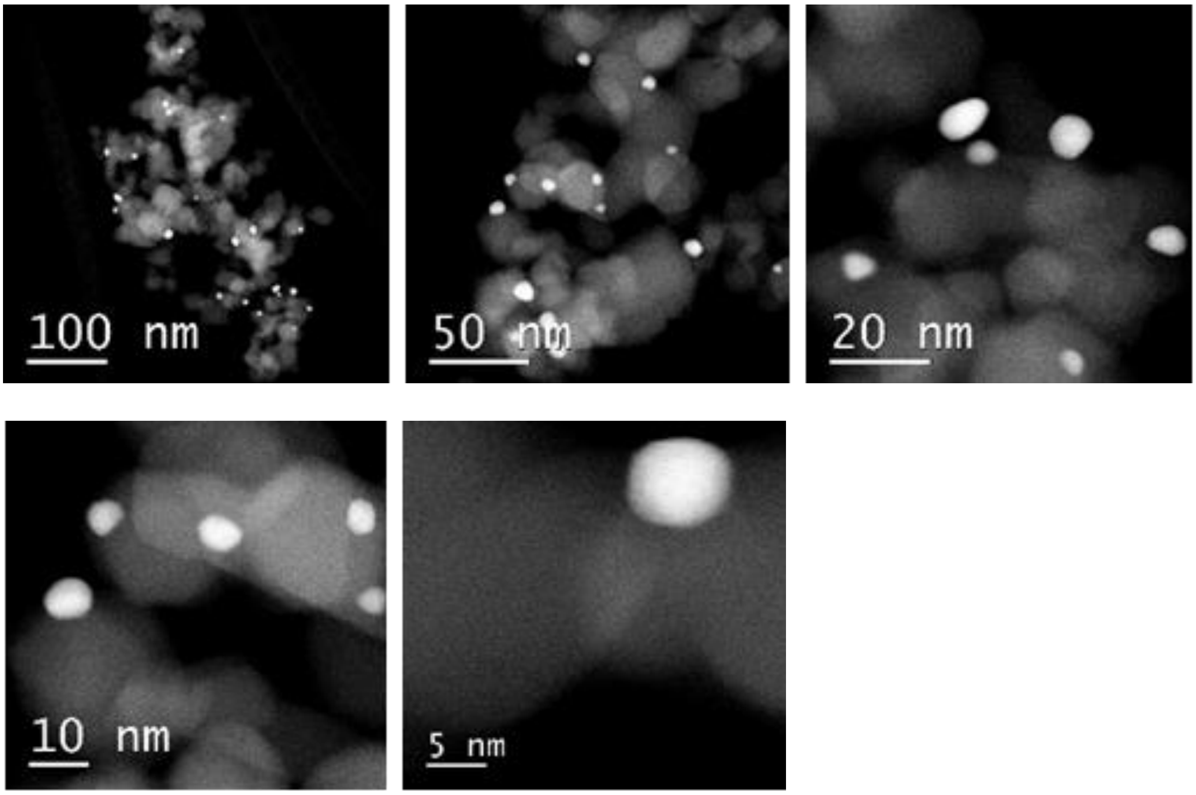

Supplementary Figure 11. AC-HAADF-STEM images of Au@SiO_2/Ti-800.

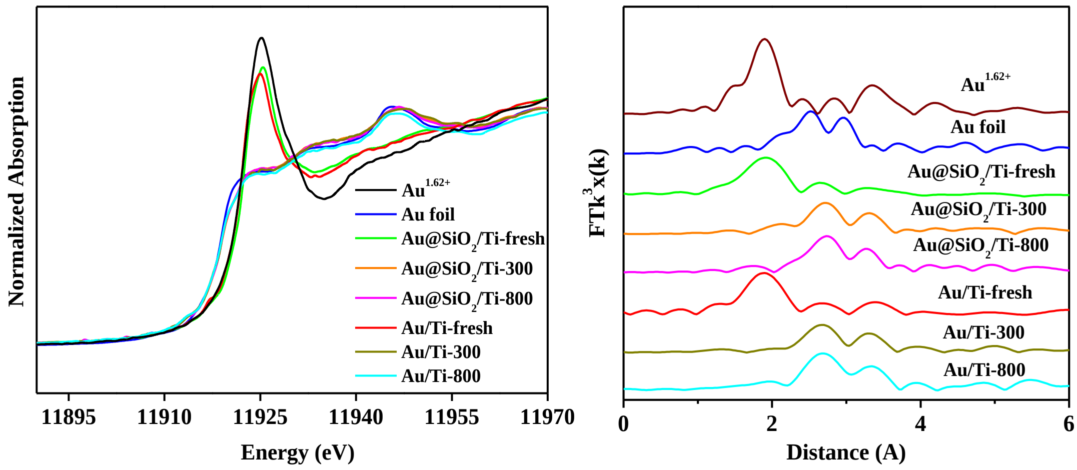

Supplementary Figure 12. Au L_3-edge XANES profiles (left) and EXAFS fitting results in R space (right) of various gold samples.

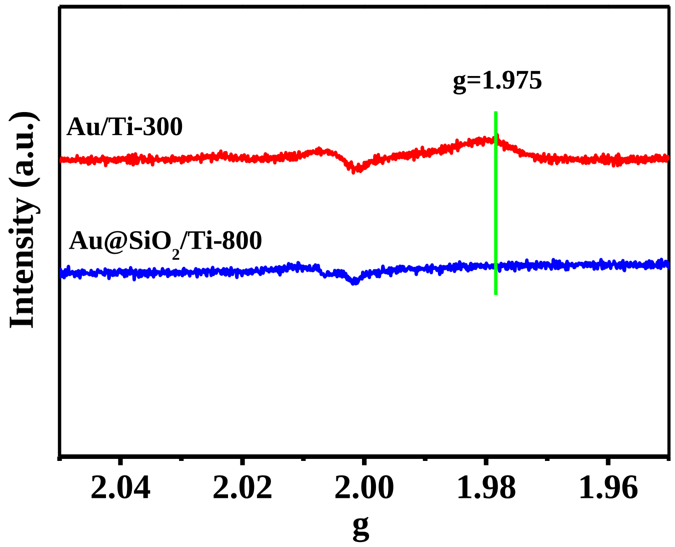

Supplementary Figure 13. EPR spectra of Au/Ti-300 and Au@SiO_2/Ti-800. g =1.975 displays the signal of Ti^{3+}.

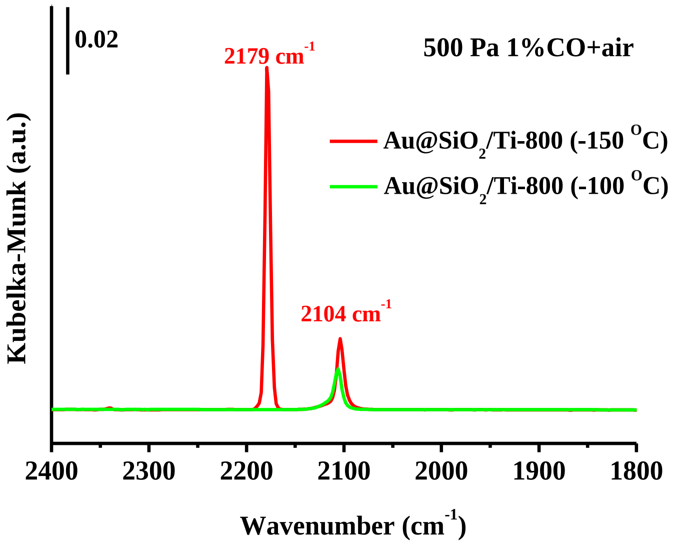

Supplementary Figure 14. The in-situ DRIFT spectra of CO adsorption at different temperatures on Au@SiO_2/Ti-800 after introducing 500 pa gas mixture of 1% CO and air.

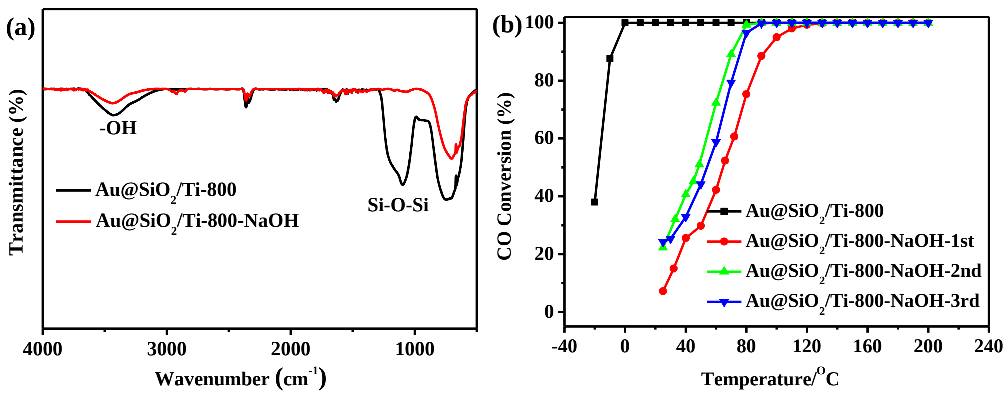

Supplementary Figure 15. (a) FT-IR spectra of Au@SiO_2/Ti-800 and Au@SiO_2/Ti-800-NaOH. (b) CO conversion versus reaction temperature on Au@SiO_2/Ti-800 and Au@SiO_2/Ti-800-NaOH with 3 cycles. Conditions: 150 mg of catalyst, 50 mL min^{-1} of 1 vol.% CO + 20 vol.% O_2 and balanced with N_2, and the space velocity of 20 L $g_{cat.}^{-1}$ h^{-1}.

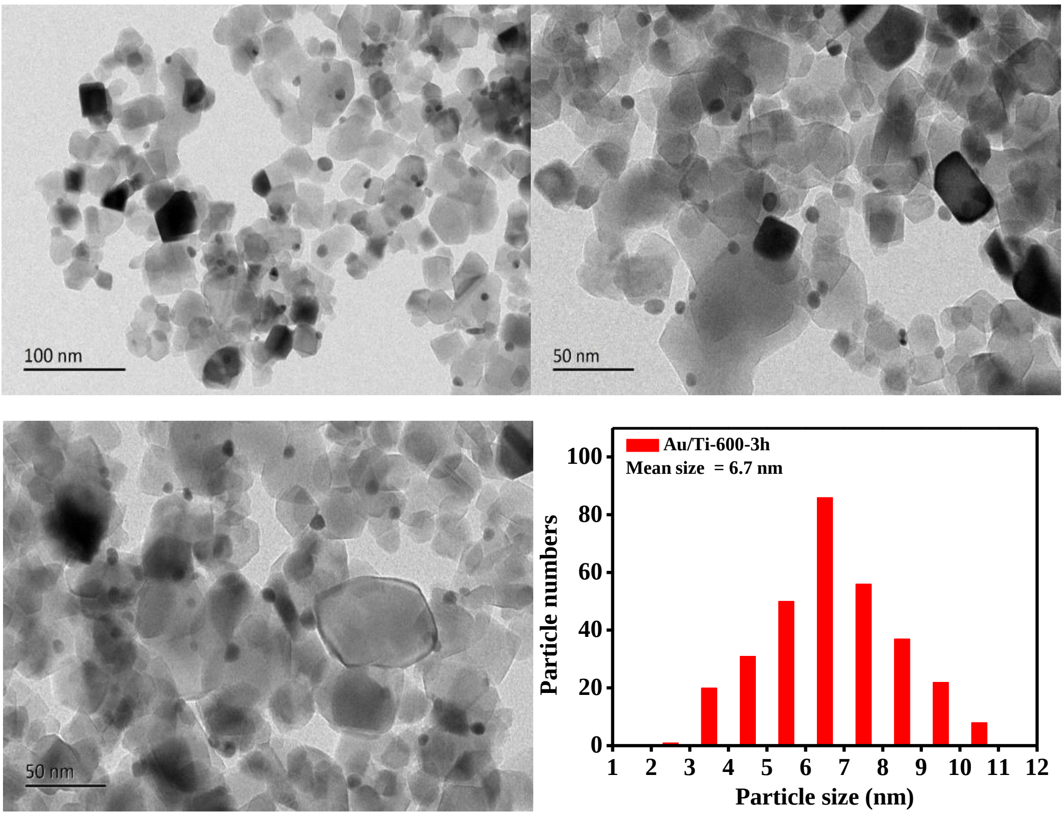

Supplementary Figure 16. TEM images of Au/Ti-600-3h and corresponding size distributions of gold particles.

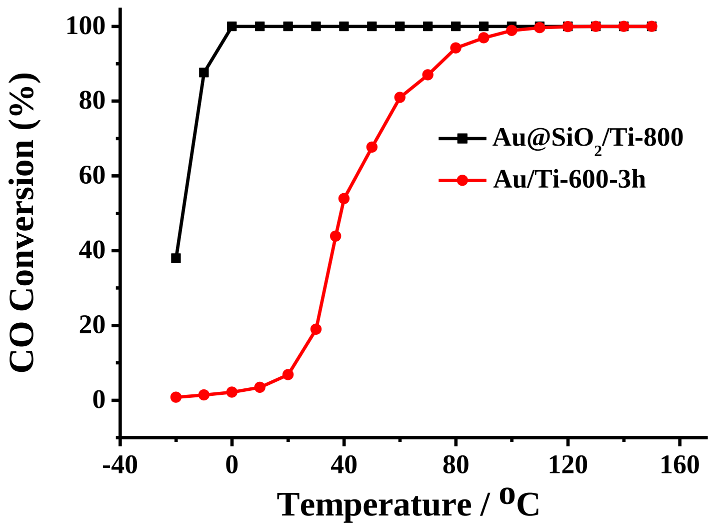

Supplementary Figure 17. The CO oxidation activity of Au@SiO_2/Ti-800 and Au/Ti-600-3h. Conditions: 150 mg of catalyst, 50 mL min^{-1} of 1 vol.% CO + 20 vol.% O_2 and balanced with N_2, and the space velocity of 20 L $g_{cat.}^{-1}$ h^{-1}.

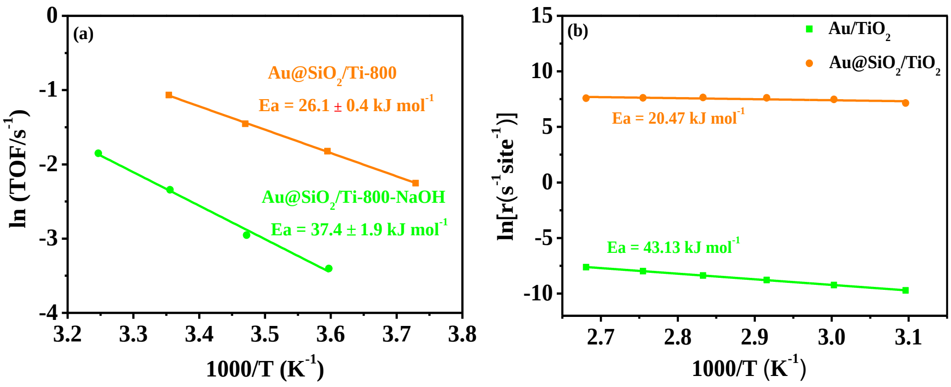

Supplementary Figure 18. Arrhenius plots of (a) the experimental reaction rate ln(TOF) vs 1/T for Au@SiO_2/Ti-800 and Au@SiO_2/Ti-800-NaOH and (b) the theoretical reaction rate ln(r) vs 1/T for Au/TiO_2 and Au@SiO_2/TiO_2.

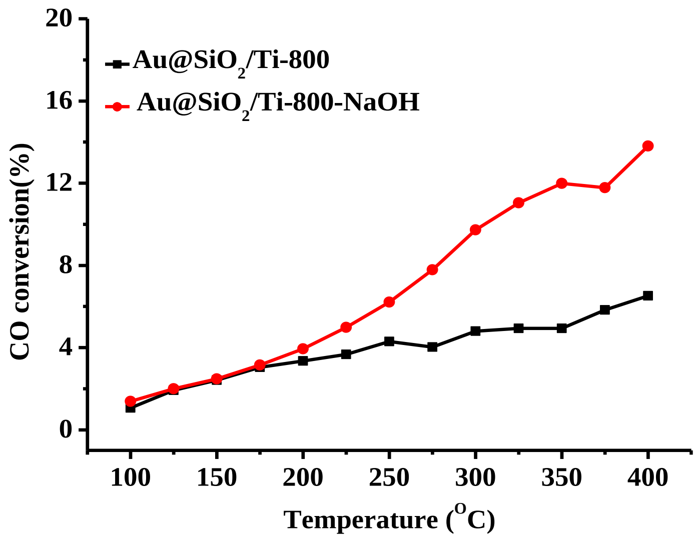

Supplementary Figure 19. Water gas shift activity of Au@SiO_2/Ti-800 and Au@SiO_2/Ti-800-NaOH. Conditions: 100 mg of catalyst, 30 mL min^{-1} of 10 vol.% H_2O + 2 vol.% CO and balanced with N_2, and the space velocity of 18 L $g_{cat.}^{-1}$ h^{-1}.

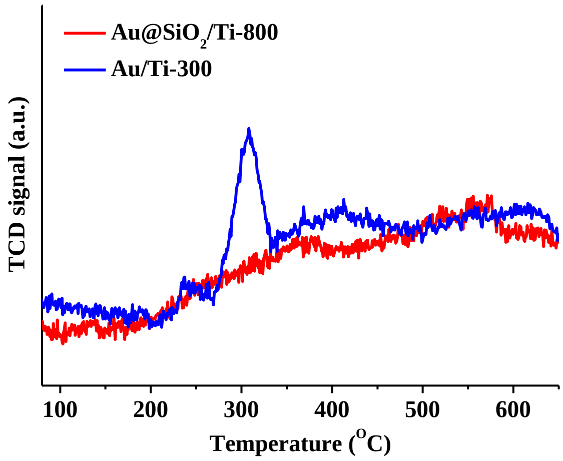

Supplementary Figure 20. **H_2-TPR of Au/Ti-300 and Au@SiO_2/Ti-800.**

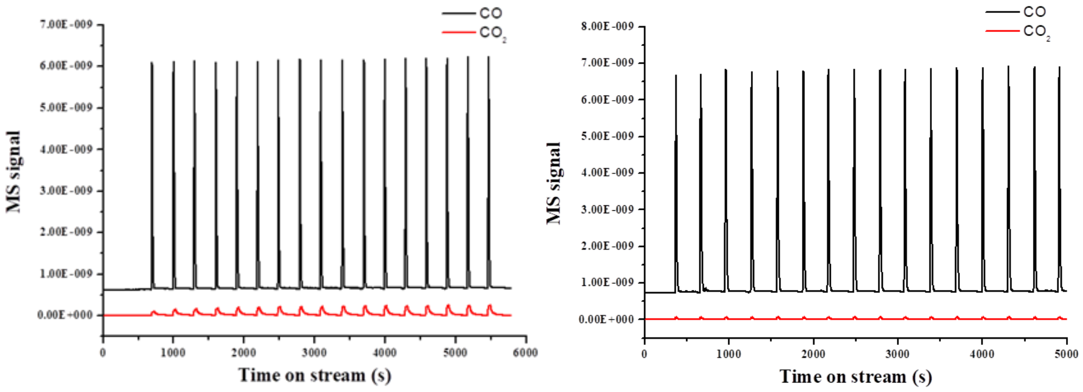

Supplementary Figure 21. CO pulse reactions of Au/Ti-300 (left) and Au@SiO_2/Ti-800 (right) at 50 ℃.

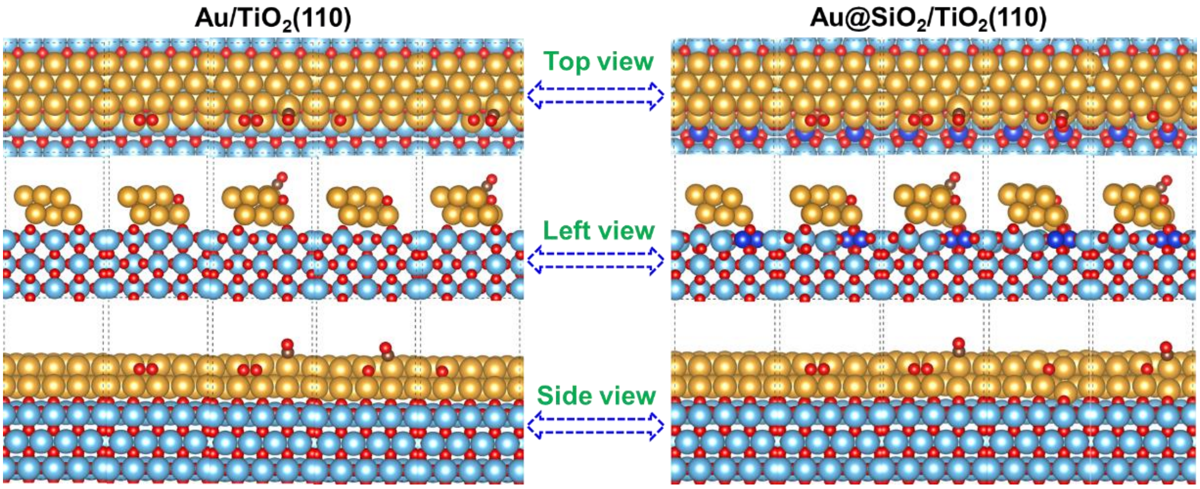

Supplementary Figure 22. The adsorbed structures of CO oxidation reactions on Au/TiO_2 (110) and Au@SiO_2/TiO_2 (110) models. The gold, red, cyan, brown and blue balls are Au, O, Ti, C and Si atoms, respectively.

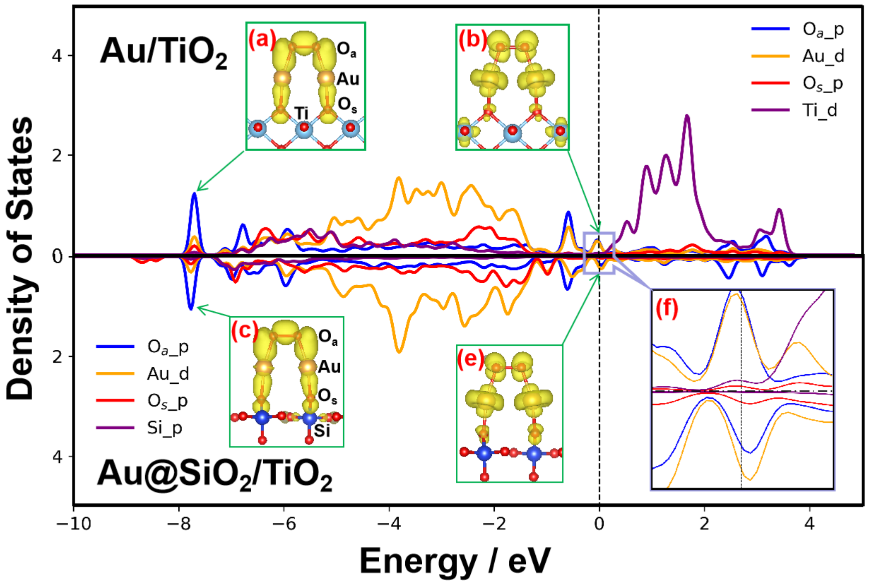

Supplementary Figure 23. The calculated partial density of states of adsorbed O_2 on Au/TiO_2 (upper) and Au@SiO_2/TiO_2 (bottom). The inserted figures a-e show the partial charge density of some important bands and figure f is enlarged view of partial density of states at Fermi level.

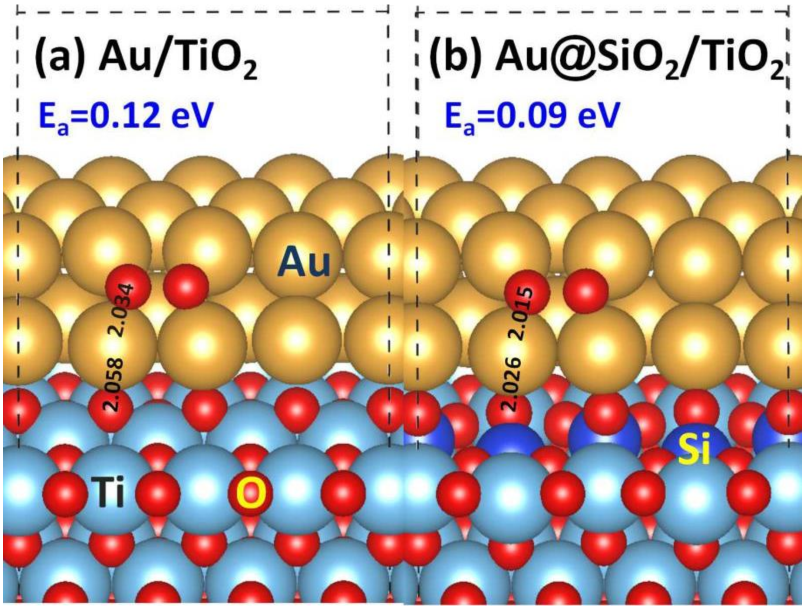

Supplementary Figure 24. The transition state structures of O_2 direct dissociation on Au/TiO_2 (110) and Au@SiO_2/TiO_2 (110).

Supplementary Table 1. The electronegativity of the different cation in oxides.

Oxides	Electronegativity
MgO	6.55
ZnO	8.25
FeO	9.15
CuO	9.50
CeO_2	10.08
Al_2O_3	11.27
ZrO_2	11.97
Fe_2O_3	12.81
TiO_2	13.86
MnO_2	13.95
SiO_2	17.10

Supplementary Table 2. The ICP results of Au and Si in different gold catalysts.

Catalyst	Au (wt%)	Si (wt%)
Au/Ti-fresh	0.98	-
Au@SiO_2/Ti-fresh	0.93	2.09
Au@SiO_2/Ti-800-NaOH	1.00	0.13

Supplementary Table 3. The surface area of different gold catalysts.

Catalysts	Surface area (m^2/g)
Au/Ti-300	54.4
Au/Ti-800	10.1
Au@SiO_2/Ti-300	216.5
Au@SiO_2/Ti-800	60.3

Supplementary Table 4. The percentage of metallic gold and cationic gold in different gold catalysts calculated by the XPS results.

Catalyst	Metallic gold (%)	Positively charged gold (%)	Au^+/Au^0
Au/Ti-fresh	79 (83.2 eV)	21 (83.8 eV)	0.27
Au/Ti-300	82 (83.2 eV)	18 (83.8 eV)	0.22
Au/Ti-800	100 (83.2 eV)	0.0 (83.8 eV)	0
Au@SiO_2/Ti-fresh	21 (83.6 eV)	79 (83.8 eV)	3.76
Au@SiO_2/Ti-300	25 (83.6 eV)	75 (83.8 eV)	3.00
Au@SiO_2/Ti-800	82 (83.5 eV)	18 (83.8 eV)	0.22

Supplementary Table 5. TOF values of various gold catalysts in this work and of the most sintering resistant Au catalysts reported in literatures.

Catalysts	Au loading (wt%)	D_{Au} [(a)] (nm)	TOF [(b)] (s^{-1})	Temperature (°C)	Supplementary References
Au/Ti-800	0.98	20.4 ± 4.2	0.02	25	this work
Au@SiO_2/Ti-800	0.93	6.4 ± 1.4	0.31	25	this work
Au@SiO_2/Ti-800-NaOH	1.0	7.8 ± 1.4	0.13	25	this work
Au/(SiO_2-TiO_2)-700	2.2	6.5	0.19	64	Ref 1
Au/(Fe_2O_3-HAP)-600	3.2	4.5	0.02	24	Ref 2
Au/ZnO-600	1.94	6.3	~ 0.07	100	Ref 3
Au/(ZrO_2-SiO_2)-800	5.7	8.7 ± 3.9	0.02	20	Ref 4
Au/(TiO_2-SiO_2)-800	2.1	3.5 ± 1.6	0.18	20	Ref 4
Au/TH-800	1.0	4.9 ± 1.0	0.12	25	Ref 5
Au/TiO_2-RR2Ti [(c)]	1.0	4.1 ± 2.2	0.38	25	Ref 5
Au/TiO_2-WGC [(d)]	1.47	3.8 ± 0.8	0.39	25	Ref 5

(a) Measured by TEM; (b) The gold dispersion was calculated according to $D = 1/d_{Au}$, where d means diameter; (c) Provided by Haruta Gold Inc.; (d) Provided by World Gold Council.

Supplementary Table 6. Au L_3-edge EXAFS fitting results (*R*: distance; *CN*: coordination number; σ^2: Debye-Waller factor; ΔE_0: inner potential correction) of different gold catalysts.

sample	shell	*R* (Å)	*CN*	ΔE_0 (eV)	σ^2 (Å^2)
Au@SiO_2/Ti-fresh	Au-O	1.95 ± 0.01	3.2 ± 0.5	9.2 ± 0.9	0.004 ± 0.002(O)<br>0.008 ± 0.001(Au)
	Au-Au	2.88 ± 0.02	2.2 ± 0.7		
Au@SiO_2/Ti-300	Au-Au	2.86 ± 0.01	8.4 ± 1.3	7.2 ± 0.9	
Au@SiO_2/Ti-800	Au-Au	2.86 ± 0.01	9.2 ± 1.8	7.2 ± 0.9	
Au/Ti-fresh	Au-O	1.95 ± 0.02	3.4 ± 1.0	9.2 ± 0.9	
	Au-Au	2.90 ± 0.06	3.0 ± 2.6		
Au/Ti-300	Au-Au	2.84 ± 0.01	7.4 ± 1.4	7.2 ± 0.9	
Au/Ti-800	Au-Au	2.87 ± 0.01	11.8 ± 2.2	7.2 ± 0.9	

Supplementary Table 7. The chlorine ion content and the ratio of chlorine ion to Au for different catalysts.

Sample	Cl^- content (ppm)	Cl^-/Au ratio
Au@SiO_2/Ti-800	3	0.0003
Au@SiO_2/Ti-800-NaOH	<3	<0.0003

Supplementary Table 8. Reaction steps, reaction heat, reaction barrier, calculated reaction rate and degree of thermodynamic rate control (X_{RC}) of CO oxidation on Au/TiO_2 catalysts.

No	Reaction steps	ΔE (eV)	E_a (eV)	Rate (s^{-1} $site^{-1}$)	X_{RC}
1	CO(g)+*↔CO*	-0.60	0.00	$3.01x10^{-7}$	0.00
2	O_2(g)+*↔O_2*	0.38	0.38	$1.51x10^{-7}$	0.00
3	O_2*+*↔2O*	-0.49	0.12	$1.51x10^{-7}$	1.00
4	CO*+O*↔CO_2(g)+2*	-2.60	0.00	$3.01x10^{-7}$	-5.99×10^{-9}

Note: Calculated coverage: CO*: 0.711; O_2*: 1.49×10^{-17}; O*: 7.43×10^{-20}; *:0.289.

Supplementary Table 9. Reaction steps, reaction heat, reaction barrier, calculated reaction rate and degree of thermodynamic rate control (X_{RC}) of CO oxidation on Au@SiO_2/TiO_2 catalysts.

No	Reaction steps	ΔE (eV)	E_a (eV)	Rate (s^{-1} $site^{-1}$)	X_{RC}
1	CO(g)+*↔CO*	-0.68	0.00	0.702	-3.81×10^{-5}
2	O_2(g)+*↔O_2*	-0.08	0.00	0.351	5.17×10^{-7}
3	O_2*+*↔2O*	-0.29	0.09	0.351	1.00
4	CO*+O*↔CO_2(g)+2*	-2.39	0.00	0.702	3.79×10^{-13}

Note: Calculated coverage: CO*: 0.987; O_2*: 2.10×10^{-10}; O*: 1.25×10^{-13}; *:1.34×10^{-2}.

Supplementary References

1. Zhu, H. G., Ma, Z., Overbury, S. H. & Dai, S. Rational design of gold catalysts with enhanced thermal stability: post modification of Au/TiO_2 by amorphous SiO_2 decoration. *Catal. Lett.* 116, 128-135 (2007).
2. Zhao, K., Qiao, B., Wang, J., Zhang, Y. & Zhang, T. A highly active and sintering-resistant Au/FeOx-hydroxyapatite catalyst for CO oxidation. *Chem. Commun.* **47**, 1779-1781 (2011).
3. Liu, J., Qiao, B., Song, Y., Huang, Y.D. & Liu, J. Hetero-epitaxially Anchoring Au Nanoparticles onto ZnO Nanowires for CO Oxidation. *Chem. Commun.* **51**, 15332-15335 (2015).
4. Puértolas, B. *et al.* High-temperature stable gold nanoparticle catalysts for application under severe conditions:the role of TiO_2 nanodomains in structure and activity. *ACS Catal.* **5**, 1078-1086 (2015).
5. Tang, H. *et al.* Ultrastable hydroxyapatite/titanium-dioxide-supported gold nanocatalyst with strong metal–support interaction for carbon monoxide oxidation. *Angew. Chem. Int. Ed.* **55**, 10606-10611 (2016).
